# High‐Fat and Low‐Carbohydrate Dietary Environments Are Linked to Reduced Idiopathic Epilepsy Incidence and Prevalence

**DOI:** 10.1002/acn3.70017

**Published:** 2025-02-25

**Authors:** Duan Ni, Alistair Senior, David Raubenheimer, Stephen J. Simpson, Ralph Nanan

**Affiliations:** ^1^ Sydney Medical School Nepean The University of Sydney Sydney New South Wales Australia; ^2^ Nepean Hospital Nepean Blue Mountains Local Health District Sydney New South Wales Australia; ^3^ Charles Perkins Centre The University of Sydney Sydney New South Wales Australia; ^4^ School of Life and Environmental Sciences The University of Sydney Sydney New South Wales Australia; ^5^ Sydney Precision Data Science Centre The University of Sydney Sydney New South Wales Australia

**Keywords:** dietary environment, high‐fat low‐carbohydrate, idiopathic epilepsy, macronutrient

## Abstract

Dietary manipulations like ketogenic diets are established interventions for recalcitrant epilepsy. However, it remains unknown whether specific macronutrient exposure through dietary environments could possibly extend to primary preventive qualities, associated with changes in epilepsy disease burden (prevalence and incidence). Here, macronutrient supply, GDP, and idiopathic epilepsy disease burden data were collated from more than 150 countries from 1990 to 2018. Nutritional geometry generalized additive mixed models (GAMMs) modeling unraveled that dietary environments with high‐fat and low‐carbohydrate supplies were linked to lower epilepsy incidence and prevalence. Our analyses suggested a plausible primary preventive role of dietary manipulations for epilepsy.

## 1 Introduction

Epilepsy is one of the leading neurological disorders globally [1]. Epilepsy pathogenesis is multifactorial, and environmental factors, like alcohol use [2], represent critical risk factors in its development. Other aspects, like diet and nutrients, might also be important, as reflected by the therapeutic effects of ketogenic diets on recalcitrant epilepsy [3, 4, 5, 6]. Previous diet‐related studies mostly concentrated on the effects of individual nutrient on epilepsy treatment [3, 4, 6, 7]. In contrast, less is known regarding the primary prevention of epilepsy via nutritional and dietary modifications, as studies of this kind are intrinsically difficult to carry out at a population level across a time scale.

Here, we leveraged nutrient supplies, a proxy for diet and nutrient environments, to interrogate the potential associations between nutrient exposures and epilepsy incidence and prevalence, harnessing the powerful tool, nutritional geometry generalized additive mixed models (GAMMs), we previously conceptualized [8, 9]. We found that dietary environments, high in fat and low in carbohydrate supplies, are associated with reduced idiopathic epilepsy prevalence and incidence, indicative of its protective effects. This might extend the clinical applications of dietary manipulation for primary prevention of epilepsy.

## 2 Materials and Methods

### 2.1 Data Collection

Disease burden data (incidence and prevalence) of idiopathic epilepsy were from the Global Burden of Disease (GBD) database [10], which is defined as *recurrent, unprovoked seizures without identified underlying disease and is presumed to have genetic bases*. As reported in our previous studies [8, 9], nutrient supply data (supply of kcal of nutrient/capita/day) were obtained from the Food and Agriculture Organization Corporate Statistical Database (www.fao.org/faostat/en/#home), reflecting nutrients available for human consumption, as a close proxy of their actual dietary intake, but excluding the ones for other uses like agricultural utilization. Global gross domestic product (GDP) data (US$/capita), as an indicator of socioeconomic wealth, was from the Maddison project [11]. Countries or time points with no record were excluded, and the resulting data spanning from 1990 to 2018 covering more than 150 countries, across all continents, were further analyzed.

### 2.2 Generalized Additive Mixed Models

Details of the analyses are described in Supporting Information S1 and our previous works [8, 9, 12]. In brief, we leveraged the state‐of‐the‐art nutritional geometry GAMM for analysis. GAMM is a form of multiple regression, sharing similar assumptions to generalized linear models. GAMMs account for the nonlinear terms as nonparametric smoothed functions, often in the form of splines, and provide a flexible manner to estimate the nonlinear associations. These nonlinear and interactive effects are gaining more and more interest in nutritional research, with growing evidence highlighting their implications in multiple aspects of health and diseases [8, 9, 13], emphasizing the significance of multidimensional thinking in nutritional research.

Here, an array of GAMMs was utilized to analyze the impacts of nutrient supplies on epilepsy disease burden, and the individual country from which the data originated was modeled with a random effect, accounting for some confounders like ethnic and genetic differences among countries. Importantly, GAMMs could also adjust for potential confounders like GDP as a proxy for socioeconomic status, and time, which was achieved in a similar way to standard linear regression used in classic epidemiological study [13]. In this context, GAMMs with multiple variables consider all combinations of the individual, additive, and interactive effects for parameters like macronutrient supply, year, and GDP data. Modeling outcomes were evaluated using Akaike information criterions [14].

GAMMs' results are interpreted via visualization onto the multidimensional nutritional space, a common platform for nutritional geometry analysis, which deciphers the impacts from multi‐parameters (e.g., different macronutrients) simultaneously, rather than the traditional simple single regression/correlation. Details for their interpretation are described in Supporting Information S1 and in our previous work [8, 9, 12, 13, 15].

## 3 Results

**FIGURE 1:**
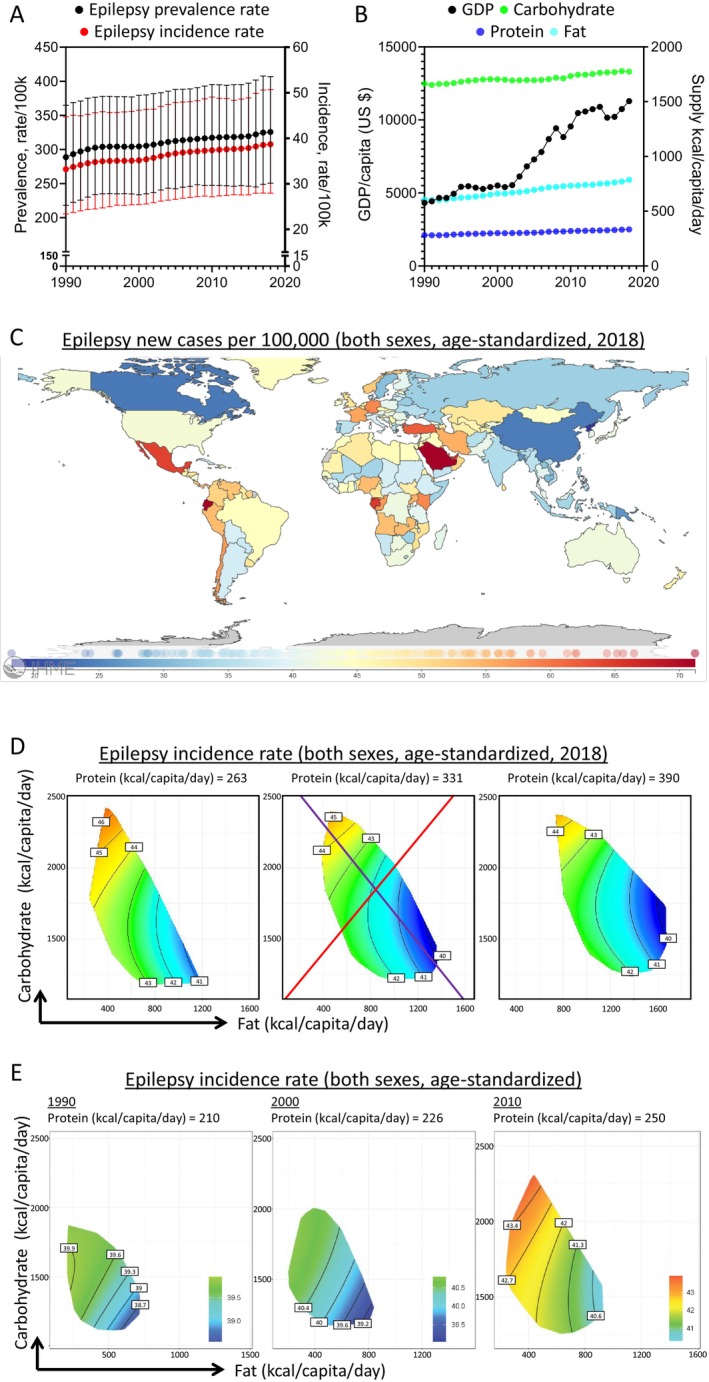
Global associations of macronutrient supplies and idiopathic epilepsy disease burden. (A) Global age‐standardized prevalence (black) and incidence (red) of idiopathic epilepsy of both sexes as functions of years. (B) Global gross domestic product (GDP) per capita (in US dollars, black), and macronutrient supplies (carbohydrate: green, protein: blue, fat: cyan) as functions of years. (C) A global overview of the age‐standardized idiopathic epilepsy incidence of both sexes in 2018. (D) Modeled effects of macronutrient supplies on age‐standardized idiopathic epilepsy incidence rate of both sexes, with 2018 results shown as representative (see Supporting Information S1 for statistics and interpretations). (E) Modeled effects of macronutrient supplies on age‐standardized idiopathic epilepsy incidence rate of both sexes in 1990 (left), 2000 (middle), and 2010 (right).

As in Figure 1A, globally, epilepsy prevalence and incidence steadily increased from 1990 to 2018. This is accompanied by concurrent increases in global GDP and correlated changes in nutritional supply landscapes, with the most striking increase in fat supply (Figure 1B, Figure S1). Epilepsy incidence exhibits a heterogeneous global distribution, affecting most countries. In 2018, there were 159 countries with data records, and the highest incidence was reported in Saudi Arabia, while the lowest in Kiribati (Figure 1C).

To interrogate the potential associations between epilepsy disease burden and nutrient supplies, a series of GAMMs were explored, which included combinations of factors including nutrient–nutrient interactions, as well as their changes over time and impacts from socioeconomic status. A model considering the interactions between macronutrient supplies and GDP, with an additive effect of time, was favored (Tables S1 and S3), illustrating the interactive effects of macronutrient supplies and socioeconomic status on epilepsy disease burden.

The results from 2018 are presented in Figure 1D as an example. This was the most recent year with relatively comprehensive data coverage. The modeled associations between macronutrient supplies and epilepsy incidence were presented as response surfaces mapped onto macronutrient supply plots. We focused on the effects of fat (*x*‐axis) and carbohydrate (*y*‐axis) supplies. Protein supply was held at 25% (low), 50% (median), and 75% (high) quantiles of global supply. Within the modeling surfaces, red represented higher, while blue denoted lower epilepsy incidences.

Our modeling found that carbohydrate supply was strongly correlated with an increased epilepsy incidence, while fat supply had the opposite association after accounting for total energy supply (Figure 1D, Tables S1 and S2). This is visualized via the purple isocaloric line. Along this vector, the total nutrient energy supply was held constant, and carbohydrate was isocalorically replaced with fat. A higher fat:carbohydrate ratio was linked to lower epilepsy burden, with the lowest epilepsy incidence found in environments with high‐fat but low‐carbohydrate supplies (bottom right). Across increasing quantiles of protein supplies, there was only a mild change in epilepsy incidence, suggesting an insignificant impact from protein. Epilepsy incidence appeared similar with changes in the total energy supply from macronutrients because along the red radial, altering the total energy supply but keeping the fat:carbohydrate ratio unchanged minimally impacted epilepsy disease burden.

**FIGURE 2:**
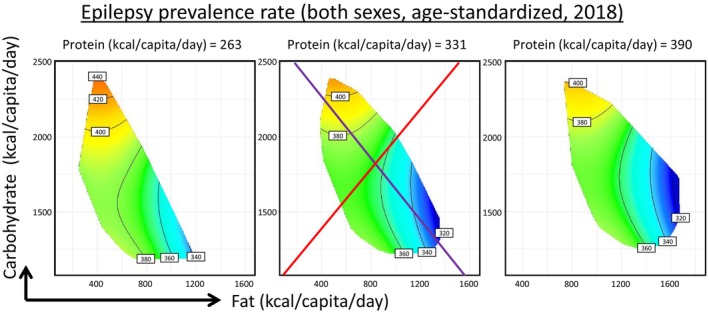
Modeled effects of macronutrient supplies on age‐standardized idiopathic epilepsy prevalence rates of both sexes, with 2018 results shown as representative.

Our modeling took into consideration the time effects, and the associations between carbohydrate supply and higher epilepsy burden were consistent across various time points (Figure 1D,E). We have also performed sensitivity tests by excluding countries with the highest (United Arab Emirates) or lowest (Democratic People's Republic of Korea) epilepsy incidences, which showed consistent results (Figure S2), highlighting the robustness of our analyses. Similarly, carbohydrate supply was also associated with increased epilepsy prevalence, while fat supply had the opposite effect (Figure 2, Tables S3 and S4). These associations were independent of sex (Figure S3A,B). Notably, further analyses sub‐dividing plant‐ and animal‐based fats revealed that they were similarly linked to epilepsy incidence (Figure S3C).

## 4 Discussion

Our work represents the first study to correlate idiopathic epilepsy disease burden with global nutritional environments, as reflected by nutrient supplies. Our analyses unveil a potential beneficial role of fat for epilepsy prevention. This has been adjusted for its plausible interactions with other macronutrients, total energy supply, and socioeconomic status, using the cutting‐edge GAMM approach [8, 9, 16]. Such systematic analysis represents the most comprehensive population‐level study in this regard to our knowledge, showcasing that increased fat supply is linked to reduced epilepsy incidence and prevalence, particularly coupled with decreased carbohydrate supply, suggesting that dietary environments high in fat but low in carbohydrate might exhibit protection against epilepsy development.

Our analyses were based on nutrient supplies, a proxy of the dietary and nutrient environment. Validation with more detailed dietary and nutritional data would be warranted. Also, it remains to be interrogated at which developmental stage across the lifespan exposure to a high‐fat, low‐carbohydrate environment will be the most beneficial for epilepsy prevention. Future cohort‐ or population‐based diet and nutrient studies might shed more light on these aspects and also evaluate the potential adverse effects [17].

Of note, despite the strong associations between dietary environment and epilepsy disease burden, the exact causality remains to be elucidated. There is a previous report on children with epilepsy exhibiting a preference for fat over carbohydrate in their diets. This might also partly explain our modeling findings [18].

Mechanistically, a recent murine study reported that ketone body β‐hydroxybutyric acid is the main contributor to the therapeutic effect of high‐fat, low‐carbohydrate ketogenic diets in epilepsy [5]. This is also supported by a clinical study reporting the negative association between circulating ketone body levels and seizure frequency in children with medically intractable epilepsy receiving a ketogenic diet [19]. Therefore, it would be of interest to inspect which threshold levels of ketosis are required for epilepsy treatment or prevention. In addition, a high‐fat, low‐carbohydrate diet is likely to influence the gut–brain axis [20] and immunometabolism [21], which are also critical for the physiology and pathology of the CNS system.

One of the limitations of our study lies in the complexities in idiopathic epilepsy, with its multifactorial pathogenesis nature and changing diagnosis and reporting criteria across countries over time. As defined by GBD, idiopathic epilepsy refers to cases without identified underlying disease and is presumed to have some genetic basis. This at least excludes epilepsy cases of other causes like infection or trauma [10]. Our modeling partially accounted for the differences across countries regarding ancestral and genetic backgrounds, as well as correcting for GDP as a reflection of socioeconomic status, a critical factor impacting the affordability of nutrient fat, the key component in ketogenic diets. Still, further in‐depth studies are needed to thoroughly decipher other confounders for idiopathic epilepsy. This would include investigation into the interplay between genetic and environmental factors, specifically diets and nutrients. Additionally, our modeling has accounted for the time factor, showing a consistent pattern across various time points, highlighting the robustness of our results, despite the possible changes in disease reporting criteria. On the other hand, albeit comprehensive data for food processing and diet quality are still lacking on a global scale. Such data would add value to dissect the plausible impacts from nutrient quality, determined by the degree of food processing.

Together, our ecological analyses have revealed a robust association between a nutrient environment with high‐fat and low‐carbohydrate supplies and decreased epilepsy incidence and prevalence. This might inform future nutrient‐based epilepsy prevention.

## Author Contributions

Concept and design: Duan Ni and Ralph Nanan. Acquisition, analysis, and interpretation of data: Duan Ni, Alistair Senior, David Raubenheimer, Stephen J. Simpson, and Ralph Nanan. Drafting of the manuscript: Duan Ni and Ralph Nanan. Critical revision of the manuscript for important intellectual content: All authors.

## Ethics Statement

The authors have nothing to report.

## Conflicts of Interest

The authors declare no conflicts of interest.

## Supporting information


Data S1:

## Data Availability

All data used in the present study are publicly available as described in Materials and Methods in Supporting Information.
