# Supplementary material for: High‐Fat and Low‐Carbohydrate Dietary Environments Are Linked to Reduced Idiopathic Epilepsy Incidence and Prevalence
Source: Ann Clin Transl Neurol. 2025 Feb 25;12(5):1077–81. doi: 10.1002/acn3.70017 (PMC12093339; doi:10.1002/acn3.70017)
Supplement: Supplementary file 1 — Data S1: [file ACN3-12-1077-s001.pdf]

Supplementary Information

Interpretation of modelling surfaces

Details for modelling interpretation can be referred to previous publications[1-3]. In brief, using RStudio (v4.2.2.), epilepsy disease burden data were analyzed utilizing generalized additive mixed models (GAMMs)[4, 5]. Results from analysis of the supplies of three macronutrients were shown as response surfaces based on nutrient axes for three macronutrients, protein, carbohydrate and fat. For analysis of different fat types, results were plotted on response surfaces with plant-based fat as the *x*-axis and animal-based fat as the *y*-axis. The sums of carbohydrate and protein supplies were held at 25%, 50% and 75% of the global data.

Within the response surfaces, red means higher values, and blue implies lower. Along the black contour lines, the modelled values are constant and the numbers on them denote the magnitude of the parameters. The purple line is an isocaloric vector, along which the total energy supply from macronutrients is unchanged but fat is isocalorically substituted with carbohydrate. The food rail is depicted by the red line. Carbohydrate:fat ratio is held constant along it, while the total energy supply is altered.

Statistics for the modelling analysis are shown in Supplementary Tables. When the modelling analyses are significant, effects of macronutrient supplies on the modelled values (i.e., disease burden) can be deduced based on the modelling surfaces.

Supplementary Methods

Data collection and processing

Data collation and processing followed the workflow described previously[1, 3]. Epilepsy data was extracted from the Global Burden of Disease Study 2019. As described[1], macronutrient supply and gross domestic product (GDP) data were from the Food and Agriculture Organization Corporate Statistical Database (FAOSTAT, www.fao.org/faostat/en/#home) and the Maddison project[6] respectively.

Countries or time points with no data record were excluded and the resulting data spanning from 1990 to 2018 covering more than 150 countries, covering all continents, were further analyzed with R.

Generalized additive mixed models (GAMMs)

Details of the models were described in[1, 3]. In brief, GAMMs[3,4] were run to model the changes of epilepsy burden over time and disentangle the influences from macronutrient supply and GDP. Overall, GAMMs share similar assumptions to generalized linear models. They account for the nonlinear terms as nonparametric smoothed functions, often in a form of spline, and provide a flexible manner to estimate the nonlinear associations. All analyses were run using the *mgcv* package and its "gam" function[4, 5]. All models took into account the countries that the data were from as random effects. The gamma parameter, implying the smoothing degrees of the modelled effects, was calculated as $\log(n)/2$, and n is the number of combinations for countries and years with our dataset. A Gaussian family with log-link function was used for modelling.

A series of different predictor variables and their different combinations and a null model where only the random effects from countries are considered are compared. Models with multiple variables consider all combinations of the individual, additive and interactions among parameters like macronutrient supply, year and GDP data. Macronutrient supply was modelled as a three-dimensional spline, and year and GDP data were modelled as one-dimensional cubic-regression splines utilizing the "s()" function in *mgcv* package. The interactions between smooth terms on different scales such as macronutrient supply and year were modelled with "te()" function from *mgcv* package using tensor product smoothers.

Modelling results were evaluated using Akaike information criterions (AICs) and the model with the lowest AIC was favoured[7].

Codes for the analysis are adapted from GitHub, https://github.com/Nidane/Asthma-NutrientSupply.

Data for the analysis are deposited in GitHub, https://github.com/Nidane/Epilepsy-NutrientSupply.

Supplementary Tables

Supplementary Tables 1-4 are GAMMs estimates. For parametric terms the model estimates and associated standard errors (SE) and test statistics are presented. For non-parametric smooth terms, the estimated and reference degrees of freedom (reflected by edf, sumEDF, Ref.df) are shown as well as their test statistics. Smooth terms were fitter with either the standard smooth function “s()” or a tensor product smooth “te()” in the *mgcv* package. Tensor product smoothing was utilized where terms exist on different units (for example, nutrient supply and year). Country was included as a random effect using the smooth function “s()”. The model family and implemented link function is stated.

Supplementary Table 1. Relative fit of generalized additive mixed models (GAMMs) testing the predictors for age-standardized epilepsy incidence rate in both sexes. Gamma is the degrees of freedom inflation factor. Dev means the deviance explained. AIC = Akaike information criterion. GDP = gross domestic product. Delta is the differences between AICs of models and the minimum AIC. sumEDF reflects the degrees of freedom of the models. Macronutrient supply was modelled as a three-dimensional thin-plate spline. (related to Figure 1D)

GAM	Gamma	Dev	AIC	Delta	Weights	sumEDF	Formula
1	3.855439	94.9937	19265.04	1582.696	0	156.7595	1 + s(Country, bs="re")
2	3.855439	95.57433	18733.33	1050.987	6.04046520236329e-229	166.1167	s(protein.kcal, carb.kcal, fat.kcal, k=k_nut) + s(Country, bs="re")
3	3.855439	95.82611	18461.05	778.7025	8.07034287889806e-170	160.737	s(Year, k=10, bs="cr") + s(Country, bs="re")
4	3.855439	96.12953	18134.76	452.4134	5.75010450512984e-99	166.0875	s(GDP, k=10, bs="cr") + s(Country, bs="re")
5	3.855439	95.99348	18297.16	614.8217	3.11291746915237e-134	170.1643	s(protein.kcal, carb.kcal, fat.kcal, k=k_nut) + s(Year, k=10, bs="cr") + s(Country, bs="re")
6	3.855439	96.22109	18046.3	363.9575	9.28203611830669e-80	175.306	s(protein.kcal, carb.kcal, fat.kcal, k=k_nut) + s(GDP, k=10, bs="cr") + s(Country, bs="re")
7	3.855439	96.2293	18025.59	343.2509	2.91087367437179e-75	169.8055	s(Year, k=10, bs="cr") + s(GDP, k=10, bs="cr") + s(Country, bs="re")
8	3.855439	96.17783	18101.43	419.0882	9.91204695567263e-92	177.4561	te(protein.kcal, carb.kcal, fat.kcal, Year, bs=c("tp", "cr"), d=c(3,1), k=c(k_nut, 7)) + s(Country, bs="re")
9	3.855439	96.48861	17763.12	80.77542	2.88297907713333e-18	197.6296	te(protein.kcal, carb.kcal, fat.kcal, GDP, bs=c("tp", "cr"), d=c(3,1), k=c(k_nut, 7)) + s(Country, bs="re")
10	3.855439	96.27368	17983.38	301.0401	4.26553916109995e-66	175.1363	te(Year, GDP, k=10) + s(Country, bs="re")
11	3.855439	96.46425	17772.05	89.71024	3.30878117812915e-20	186.6639	te(protein.kcal, carb.kcal, fat.kcal, Year, bs=c("tp", "cr"), d=c(3,1), k=c(k_nut, 7)) + s(GDP, k=10, bs="cr") + s(Country, bs="re")
**12**	**3.855439**	**96.55677**	**17682.34**	**0**	**1**	**201.0077**	**te(protein.kcal, carb.kcal, fat.kcal, GDP, bs=c("tp", "cr"), d=c(3,1), k=c(k_nut, 7)) + s(Year, k=10, bs="cr") + s(Country, bs="re")**
13	3.855439	96.35707	17900.46	218.1142	4.33611343332191e-48	184.1969	te(Year, GDP, k=10) + s(protein.kcal, carb.kcal, fat.kcal, k=k_nut) + s(Country, bs="re")

Supplementary Table 2. Estimated effects of macronutrient supply by time and GDP per capita on age-standardized epilepsy incidence rate. Gaussian-GAMM, log-link function. (related to Figure 1D)

**Parametric coefficients**				
	**Estimate**	**Std. Error**	**t value**	**Pr(>|t|)**
**(Intercept)**	3.71294	0.01113	333.7	<2e-16
**Approximate significance of smooth terms**				
	**edf**	**Ref.df**	**F**	**p-value**
**te(protein.kcal,carb.kcal,fat.kcal,GDP)**	39.886	44.592	18.38	<2e-16
**s(Year)**	4.096	5.048	18.56	<2e-16
**s(Country)**	157.026	158.000	498.62	<2e-16
R-sq.(adj) = 0.964 Deviance explained = 96.6%				
GCV = 4.1155 Scale est. = 2.9379 n = 4465				

Supplementary Table 3. Relative fit of generalized additive mixed models (GAMMs) testing the predictors for age-standardized epilepsy prevalence rate in both sexes. Gamma is the degrees of freedom inflation factor. Dev means the deviance explained. AIC = Akaike information criterion. GDP = gross domestic product. Delta is the differences between AICs of models and the minimum AIC. sumEDF reflects the degrees of freedom of the models. Macronutrient supply was modelled as a three dimensional thin-plate spline. (related to Figure 1E)

GAM	Gamma	Dev	AIC	Delta	Weights	sumEDF	Formula
1	3.855439	94.99174	41350.68	1160.188	1.17032895065262e-252	156.5077	1 + s(Country, bs="re")
2	3.855439	95.24388	41138.64	948.1556	1.29013552243245e-206	165.8156	s(protein.kcal, carb.kcal, fat.kcal, k=k_nut) + s(Country, bs="re")
3	3.855439	95.18793	41180.49	990.0003	1.0572038061995e-215	160.6266	s(Year, k=10, bs="cr") + s(Country, bs="re")
4	3.855439	95.6942	40694.67	504.1767	3.3069004380407e-110	165.8899	s(GDP, k=10, bs="cr") + s(Country, bs="re")
5	3.855439	95.38353	41013.33	822.8458	2.09557225809927e-179	169.6911	s(protein.kcal, carb.kcal, fat.kcal, k=k_nut) + s(Year, k=10, bs="cr") + s(Country, bs="re")
6	3.855439	95.81011	40591.04	400.5542	1.04895520077913e-87	174.9974	s(protein.kcal, carb.kcal, fat.kcal, k=k_nut) + s(GDP, k=10, bs="cr") + s(Country, bs="re")
7	3.855439	95.80002	40592.82	402.3344	4.30727571856498e-88	170.5212	s(Year, k=10, bs="cr") + s(GDP, k=10, bs="cr") + s(Country, bs="re")
8	3.855439	95.70236	40728.9	538.4163	1.21443805985666e-117	187.2449	te(protein.kcal, carb.kcal, fat.kcal, Year, bs=c("tp", "cr"), d=c(3,1), k=c(k_nut, 7)) + s(Country, bs="re")
9	3.855439	96.11332	40305.23	114.7441	1.2123890193063e-25	199.7983	te(protein.kcal, carb.kcal, fat.kcal, GDP, bs=c("tp", "cr"), d=c(3,1), k=c(k_nut, 7)) + s(Country, bs="re")
10	3.855439	96.00499	40403.92	213.4333	4.50337432946742e-47	187.7718	te(Year, GDP, k=10) + s(Country, bs="re")
11	3.855439	96.14204	40271.01	80.5263	3.2654037777047e-18	199.2484	te(protein.kcal, carb.kcal, fat.kcal, Year, bs=c("tp", "cr"), d=c(3,1), k=c(k_nut, 7)) + s(GDP, k=10, bs="cr") + s(Country, bs="re")
**12**	**3.855439**	**96.22044**	**40190.49**	**0**	**1**	**204.818**	**te(protein.kcal, carb.kcal, fat.kcal, GDP, bs=c("tp", "cr"), d=c(3,1), k=c(k_nut, 7)) + s(Year, k=10, bs="cr") + s(Country, bs="re")**
13	3.855439	96.06201	40354.17	163.6792	2.86749926292708e-36	194.9879	te(Year, GDP, k=10) + s(protein.kcal, carb.kcal, fat.kcal, k=k_nut) + s(Country, bs="re")

Supplementary Table 4. Estimated effects of macronutrient supply by time and GDP per capita on age-standardized epilepsy prevalence rate. Gaussian-GAMM, log-link function. (related to Figure 1E)

**Parametric coefficients**				
	**Estimate**	**Std. Error**	**t value**	**Pr(>|t|)**
**(Intercept)**	5.91261	0.02423	244	<2e-16
**Approximate significance of smooth terms**				
	**edf**	**Ref.df**	**F**	**p-value**
**te(pbf.kcal,carb_prot.kcal,abf.kcal,GDP)**	42.963	48.294	21.67	<2e-16
**s(Year)**	4.275	5.258	17.45	<2e-16
**s(Country)**	157.580	158.000	458.18	<2e-16
R-sq.(adj) = 0.960 Deviance explained = 96.2%				
GCV = 640.38 Scale est. = 453.91 n = 4465				

Supplementary Figures

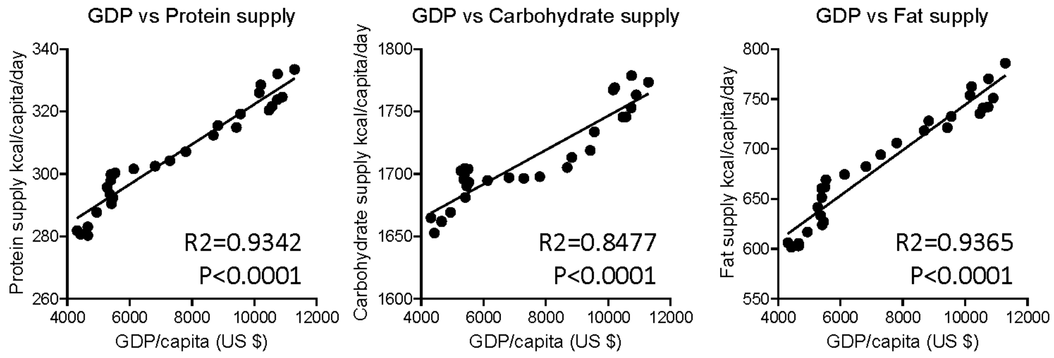

Figure S1. Correlation analysis of global gross domestic product (GDP) per capita (in US dollars) versus macronutrient supplies (protein, left; carbohydrate, middle; and fat, right).

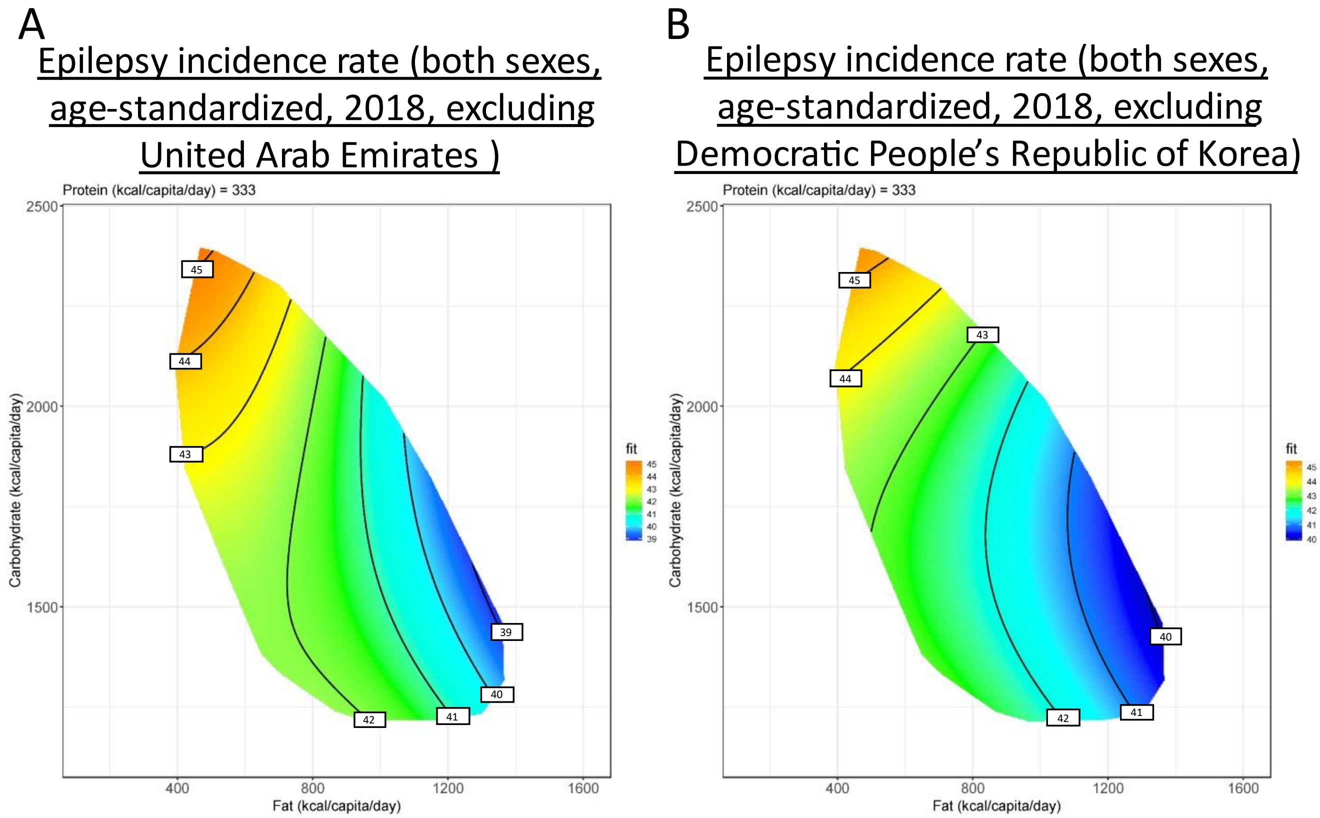

Figure S2. Modelled effects of macronutrient supplies on age-standardized idiopathic epilepsy incidence rate of both sex excluding the countries with the highest (**A.** United Arab Emirates) or lowest (**B.** Democratic People's Republic of Korea) incidence rates.

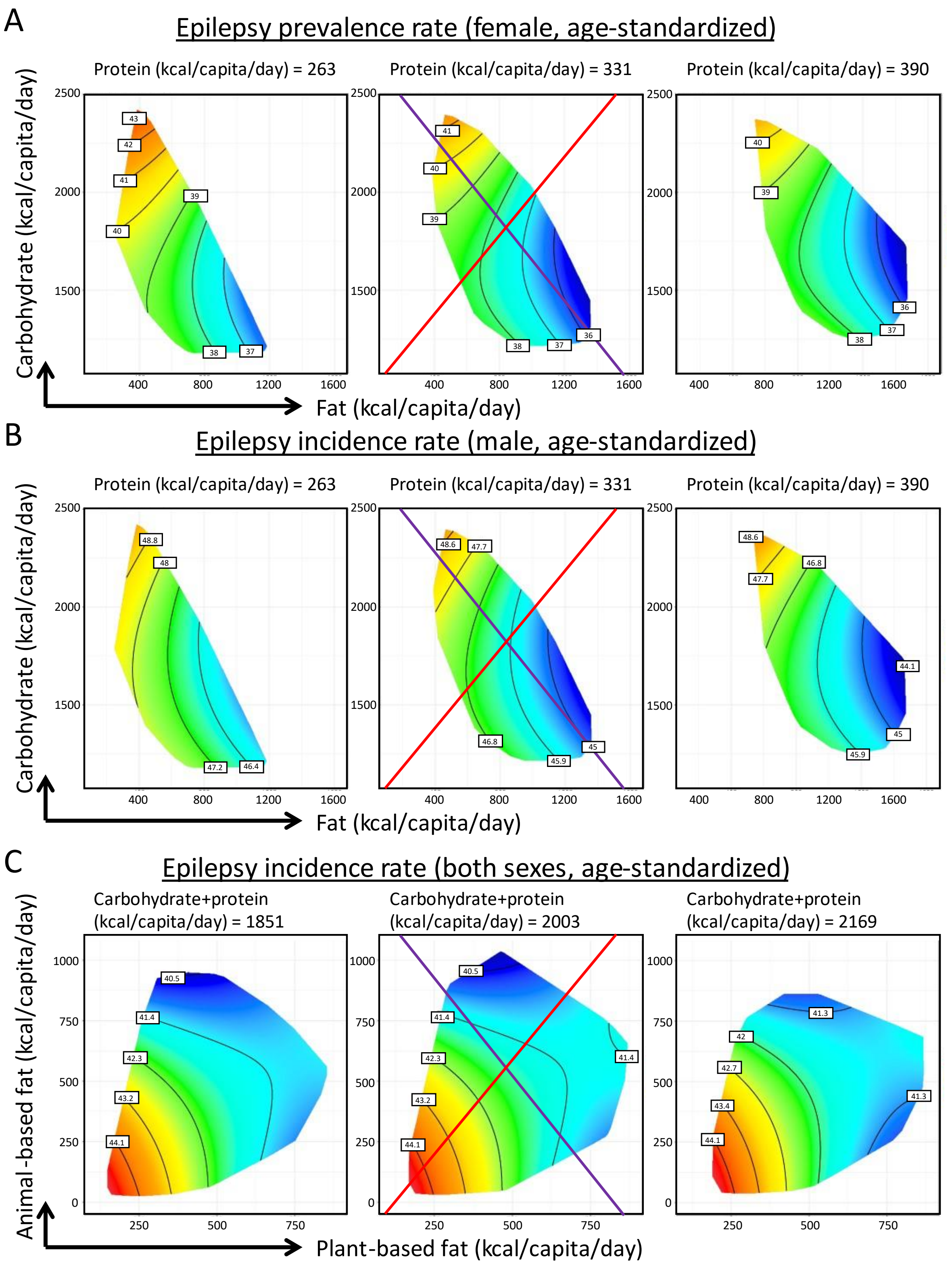

Figure S3. Modelled effects of macronutrient supplies on age-standardized epilepsy incidence rate of female (**A**), age-standardized epilepsy incidence rate of male (**B**) and predicted effects of plant-based versus animal-based fat supply on age-standardized epilepsy incidence of both sexes (**C**).

References

1. Senior AM, Nakagawa S, Raubenheimer D, Simpson SJ. Global associations between macronutrient supply and age-specific mortality. Proc Natl Acad Sci U S A 2020;117:30824-30835.
2. Wali JA, Ni D, Facey HJW, et al. Determining the metabolic effects of dietary fat, sugars and fat-sugar interaction using nutritional geometry in a dietary challenge study with male mice. Nat Commun 2023;14:4409.
3. Ni D, Senior AM, Raubenheimer D, Simpson SJ, Macia L, Nanan R. Global associations of macronutrient supply and asthma disease burden. Allergy 2024.
4. Wood SN. Generalized Additive Models: An Introduction with R: Chapman and Hall/CRC, 2017.
5. Wood SN. Fast stable restricted maximum likelihood and marginal likelihood estimation of semiparametric generalized linear models. J Roy Stat Soc B 2011;73:3-36.
6. Jutta Bolt RI, Herman de Jong, and Jan Luiten van Zanden. Rebasing 'Maddison': New income comparisons and the shape of long-run economic development. GGDC Research Memorandum: Groningen Growth and Development Center, 2018.
7. Akaike H. Information theory and an extension of the maximum likelihood principle Second International Symposium on Information Theory. Budapest1973.
